# Coupled catastrophes: sudden shifts cascade and hop among interdependent systems

**DOI:** 10.1098/rsif.2015.0712

**Published:** 2015-11-06

**Authors:** Charles D. Brummitt, George Barnett, Raissa M. D'Souza

**Affiliations:** 1 Department of Mathematics, University of California, Davis, CA 95616, USA; 2 Complexity Sciences Center, University of California, Davis, CA 95616, USA; 3 Department of Communication, University of California, Davis, CA 95616, USA; 4 Department of Computer Science, University of California, Davis, CA 95616, USA; 5 Department of Mechanical Engineering, University of California, Davis, CA 95616, USA; 6 Center for the Management of Systemic Risk, Columbia University, New York, NY 10027, USA; 7 Santa Fe Institute, Santa Fe, NM 87501, USA

**Keywords:** tipping point, regime shift, fold catastrophe, coupled systems, cascades, the Arab Spring

## Abstract

An important challenge in several disciplines is to understand how sudden changes can propagate among coupled systems. Examples include the synchronization of business cycles, population collapse in patchy ecosystems, markets shifting to a new technology platform, collapses in prices and in confidence in financial markets, and protests erupting in multiple countries. A number of mathematical models of these phenomena have multiple equilibria separated by saddle-node bifurcations. We study this behaviour in its normal form as fast–slow ordinary differential equations. In our model, a system consists of multiple subsystems, such as countries in the global economy or patches of an ecosystem. Each subsystem is described by a scalar quantity, such as economic output or population, that undergoes sudden changes via saddle-node bifurcations. The subsystems are coupled via their scalar quantity (e.g. trade couples economic output; diffusion couples populations); that coupling moves the locations of their bifurcations. The model demonstrates two ways in which sudden changes can propagate: they can cascade (one causing the next), or they can hop over subsystems. The latter is absent from classic models of cascades. For an application, we study the Arab Spring protests. After connecting the model to sociological theories that have bistability, we use socioeconomic data to estimate relative proximities to tipping points and Facebook data to estimate couplings among countries. We find that although protests tend to spread locally, they also seem to ‘hop' over countries, like in the stylized model; this result highlights a new class of temporal motifs in longitudinal network datasets.

## 1. Introduction

Sudden changes propagating among coupled systems pose a significant scientific challenge in many disciplines, yet we lack an adequate mathematical understanding of how local sudden changes spread [1]. The Earth's biosphere, for example, appears to be approaching several planetary-scale sudden changes triggered by human activity, including species extinction, desertification and lake eutrophication, which spread from one spatial patch to another [1]. That spatial spread not only poses dangers but also opportunities for detecting early warning signs [2–4]. Socioeconomic systems have examples, too: booms and busts in business cycles in different economies appear to be synchronizing because of trade, financial and other linkages [5–8]. Poverty traps at multiple scales seem to be coupled [9]. Abrupt declines in an asset price can trigger sharp declines in confidence and fire sales of other assets, as occurred in the 2007–2008 global financial crisis [10]. Protests and social uprisings appear to spread contagiously among countries, with one protest seeming to inspire others via news and social media [11,12]. The equilibrium supply and demand of a new technology that replaces an old one (such as compact discs replacing cassettes or electric cars replacing fuel cars) can change abruptly [13], and movement of people between distinct markets can facilitate adoption of the new technology [14]. In each of these examples, a system consists of distinct subsystems that (i) change suddenly between equilibria and (ii) are coupled. A mathematical understanding of these phenomena could pave the way to predicting and to steering these sudden changes.

In this paper, we take a step toward the goal of mathematically understanding how sudden changes can spread among coupled systems [1]. Our model consists of one *system*, such as the global economy or a large ecosystem, that consists of multiple *subsystems* coupled to one another; for example, economies of multiple countries are coupled by trade, while patches of an ecosystem are coupled by movement of organisms. To choose dynamics, we note that many models of the aforementioned phenomena (cited in the second column of table 1) have one or three equilibria and an S-shaped bifurcation diagram (which is equivalent to a slice of the cusp catastrophe [35]). Thus, we let each subsystem evolve according to the normal form of this catastrophe. The state of each subsystem can change suddenly when it passes a saddle-node bifurcation, one of the simplest types of ‘regime shifts’ (which are sudden changes in a system's state) [36]. Next, we introduce linear couplings between these subsystems, meaning that a change in one subsystem affects other subsystems coupled to it in proportion to that change. These couplings move the locations of the latter subsystems' bifurcations.

**Table 1.** Examples of coupled subsystems in which each subsystem undergoes sudden changes in the form of saddle-node bifurcations, in models cited in the column ‘regime shift'. The column ‘scalar quantity' describes the state of the subsystem, and it corresponds to *x*(*t*), *y*(*t*) or *z*(*t*) in the model in §2. Citations in the fourth column include empirical studies and mathematical models.

discipline	regime shift	scalar quantity	examples of couplings among subsystems
ecology	extinction due to over-harvesting [15,16]	population	diffusion among patches of an ecosystem [2,4]
economics	boom and bust in the Kaldor model of business cycles [17]	output (gross domestic product)	investment between sectors [18], trade [13] and capital flows [19] between countries can synchronize business cycles
economics	currency crisis (devaluation or, for a peg, loss of reserves) [20]	currency value	changes in macroeconomic fundamentals, sentiment, perceived riskiness, risk aversion [20] and trade [21]
economics	poverty trap [22,23]	well-being (capital, capabilities)	fractal poverty traps [9]
finance	asset price declines [24,25]	asset price	asset-to-asset contagion (a bank with a declining asset sells other assets) [26]
finance	probability of bank failure [27]	probability of bank failure	worry about institutions' creditworthiness spreads contagiously [28]
technology adoption	sudden change to new platform [13,29]	difference between supply and demand of the new platform	movement of people among distinct markets [14]
political	uprisings, revolts [30,31]	number of protestors	communication spreads inspiration, successful strategies across borders [11,12,32,33]; raising importance of identity [31] that span borders [34]

This model allows us to explore how regime shifts can synchronize and spread. Suppose one subsystem *X* ‘drives’ (i.e. affects) another subsystem *Y*, which we denote by 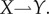 Then a regime shift in *X* can trigger one in *Y*, meaning that their regime shifts synchronize. If the driven subsystem *Y* drives a third subsystem *Z* (i.e. if 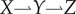), then one possible behaviour is a *cascade* of regime shifts, one triggering another like falling dominoes. Another possibility is that the ‘intermediate’ subsystem *Y* is far from its tipping point but that the others (*X* and *Z*) are close to their tipping points; then a regime shift in the driver subsystem *X* can nudge the intermediate subsystem *Y* enough to push *Z* past its tipping point but not so much that *Y* passes its tipping point. That is, a sequence of regime shifts can ‘hop’ over intermediate subsystems. This phenomenon is not observed in classic models of cascades (e.g. percolation, epidemic spreading and sandpile models).

This ‘model of many models’ abstracts from many domain-specific details. It suggests what might happen in more realistic settings. To give an example, we consider protests erupting nearly simultaneously in many countries. We first show how two sociological theories of revolutions give rise to the same S-shaped bifurcation diagram used to model the individual subsystems of our mathematical model. We also indicate how our model can generalize these sociological theories to multiple, coupled countries in a stylized way. Then we consider data on the Arab Spring, the revolts and uprisings that seemingly cascaded among countries in the Middle East and Northern Africa starting in December 2010 [12]. We explore whether protests spread locally in two networks that capture possible influence to protest, Facebook and shared borders, but we also find evidence of protests seeming to hop over countries.

Our approach differs from the many recent studies of cascades in interdependent networks [37–39], all of which model ‘interdependence’ and ‘coupling’ as occurring between pairs of nodes (individual ‘agents’) belonging to different subsystems. Instead, we consider subsystems coupled via some aggregate quantity, such as investment between sectors [18] or the fraction of people protesting in a country [30].

Much attention is paid to regime shifts in large, central nodes, such as recessions in central economies or insolvency of large banks. Our findings suggest that small changes in these central nodes (potentially triggered by a large change in a small node adjacent to it) can suffice to trigger a regime shift in a peripheral node close to its tipping point.

## 2. Normal-form model of coupled subsystems with one or two stable states

We begin by considering two subsystems *X* and *Y*, each described by a single real number, *x*(*t*) and *y*(*t*), that changes over time *t*. (Interpretations of *x*(*t*), *y*(*t*) for various contexts are given in the third column of table 1.) The subsystems evolve according to the autonomous ordinary differential equations
2.1*a*
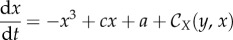
and
2.1*b*
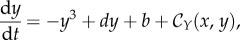
where 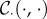 are some coupling functions (specified later), and where 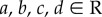 are parameters that change slowly compared with *x*(*t*), *y*(*t*), so System (1) is a *fast–slow system* [40].

Variants of System (1) have been studied in many contexts, including the double cusp catastrophe [41–43], cuspoidal nets [44,45] and coupled van der Pol oscillators [46–53] (for more information, see appendix B). Coordination games and global games in economics are similar to System (1) in that they also permit multiple equilibria, but they lack dynamics. Global games have been applied to currency crises [54], debt crises [55,56], bank runs [57,58], and riots and political change [59,60]; moreover, contagion has been studied in generalizations of these models, such as currency crises triggering more currency crises [20], bank crises triggering more bank crises [57] and currency crises triggering bank crises [61]. Here we take a catastrophe-theoretic approach [35] and emphasize the role of multiple equilibria rather than eliminate multiple equilibria, as in single-period global games.

To isolate the effect of coupling, here we focus on contagion of regime shifts in a simple setting, the singular limit in which *x*(*t*) and *y*(*t*) change arbitrarily more quickly than the ‘slow parameters’ *a*, *b*, *c*, *d*. Thus, we focus on the critical manifold, i.e. the solutions (x*, y*) to System (1) with d*x*/d*t* = d*y*/d*t* = 0.

Next, we briefly review the familiar result that, in the absence of coupling, the subsystems evolving according to equation (2.1*a*) and equation (2.1*b*) each have two saddle-node bifurcations, and then we show how coupling functions 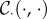 move those ‘tipping points’.

### 2.1. Uncoupled systems each undergo a cusp catastrophe

If the coupling functions 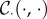 are identically zero, then subsystems *X* and *Y* are *uncoupled*, and equations (2.1*a*) and (2.1*b*) are the normal forms of the cusp catastrophe (in the special case of a minus sign on the cubic term [62, theorem (8.1)]). We chose this form to study the general effects of couplings rather than domain-specific versions of the cusp, which are topologically equivalent to the normal form in equation (2.1*a*). Hereafter, we take *c* = *d* = 1 for simplicity.

**Figure 1.**
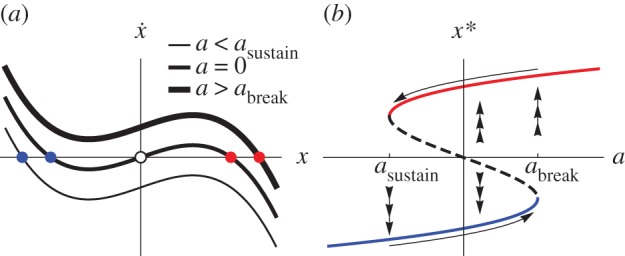
In isolation, each system has two saddle-node bifurcations. (*a*) The flow 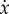 in equation (2.1*a*) has one or three equilibria, depending on the value of the parameter *a*. Filled (respectively, open) circles denote stable (unstable) equilibria. (*b*) The bifurcation diagram (the equilibria x* of (2.1*a*) as a function of *a*) is a slice of the cusp catastrophe, with two saddle-node bifurcations at values of *a* denoted by *a*_break_ and by *a*_sustain_. The solid (respectively, dashed) curves are stable (unstable) fixed points x*. Triple arrows denote the fast flow (*a*); single arrows denote a slow flow d*a*/d*t* described in the text.

If the subsystems evolving according to equation (2.1*a*) and equation (2.1*b*) are uncoupled, then both subsystems have three equilibria for certain intervals of the slow parameters *a* and *b*, as depicted in figure 1. In this case, the set of fixed points of equation (2.1*a*) undergoes two saddle-node bifurcations at values of *a* that we denote by *a*_break_ and by *a*_sustain_ (the same terminology used in [13]). Each subsystem is a classic example of *hysteresis*. For instance, if the equilibrium x* of equation (2.1*a*) is on the ‘lower stable branch’ (the blue curve in figure 1*b*), then as *a* increases past the ‘breaking point’ *a*_break_, the solution *x*(*t*) jumps to the ‘upper stable branch’ depicted by the red curve. (In other words, the subsystem passes a tipping point (undergoes a regime shift).) As the parameter *a* is slowly decreased, the large equilibrium is sustained (i.e. *x*(*t*) lies on the red curve) until *a* passes *a*_sustain_, at which point the subsystem *x*(*t*) jumps to the lower branch.

### 2.2. Master–slave with linear coupling

Next, we consider the analytically solvable case of a master–slave system with linear coupling. Specifically, subsystem *X* drives subsystem *Y* (denoted 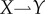) according to the coupling function 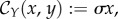 where the constant 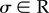 is the coupling strength. (For instance, consider unidirectional investment between sectors in the Kaldor business cycle model, as in [18], or movement of organisms from one patch of an ecosystem to another, as in [2,4].) Then equation (2.1) becomes
2.2*a*
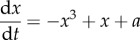
and
2.2*b*
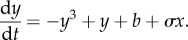
The equilibria of equation (2.2) can be obtained analytically by first solving for the equilibria x* of the master subsystem (equation (2.2*a*)), and then by using the solution(s) to calculate the equilibria y* of the slave subsystem (equation (2.2*b*)). The saddle-node bifurcations of the slave subsystem (equation (2.2*b*)) now depend on the equilibrium value(s) x* of the master subsystem (equation (2.2*a*)) and on the coupling strength *σ*; we denote the slave subsystem's bifurcations (with respect to *b*) by *b*_break_(σx*) and by *b*_sustain_(σx*). Because x* has three possible values whenever the master parameter 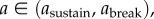 the slave subsystem has three possible values for each of its bifurcation points *b*_break_(σx*) and *b*_sustain_(σx*) whenever 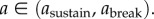


**Figure 2.**
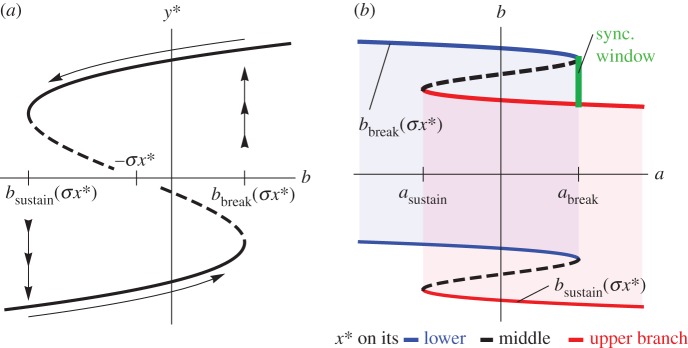
Coupling a slave subsystem to a master subsystem moves the slave subsystem's tipping points and can change them suddenly. (*a*) The bifurcation diagram of the slave subsystem shows the equilibrium of the slave subsystem y*(*b*; σx*) as a function of its slow parameter *b*. (The slave subsystem's equilibrium y* also depends on the coupling term σx* due to the influence of the master subsystem.) In this example, the master subsystem has just passed its break point *a* > *a*_break_, so the master subsystem has quickly moved to its upper stable branch of equilibria (x* > 0). Because the coupling strength *σ* > 0, the sudden shift in the master subsystem makes it easier for the slave subsystem to pass its break point [*b*_break_(σx*) < *b*_break_(0)]. (*b*) The locations of the saddle-node bifurcations of the slave subsystem (equation (2.2*b*)), denoted by *b*_break_(σx*) and by *b*_sustain_ (σx*), are one- or three-valued functions of *a*, the parameter of the master subsystem. The colours match those in figure 1: if the master subsystem's equilibrium x* lies on its lower (respectively, upper) stable branch depicted in figure 1*b*, then the bifurcation points of the slave subsystem are the blue (respectively, red) curves in (*b*). There exist three equilibria y* in the shaded blue (respectively, red) regions. Here, *σ* > 0, so the master subsystem acts to prevent the slave subsystem from crossing its break point *b*_break_(σx*) when x* < 0 and facilitates it when x* > 0. If (*a,b*) crosses the green line segment marked ‘sync. window', then the regime shifts *synchronize*: the master subsystem (equation (2.2*a*)) crosses its break point *a*_break_, causing x* to jump from a negative number to a positive number, which causes the slave subsystem (equation (2.2*b*)) to cross its break point *b*_break_(σx*).

Figure 2 shows the resulting bifurcation diagrams of the slave subsystem for *σ* = 0.1. The saddle-node bifurcations are now functions of the coupling term: *b*_•_(σx*) = *b*_•_(0)−σx*, where • is either ‘break’ or ‘sustain’, and *b*_•_(0) is the bifurcation when the subsystems are uncoupled (*σ* = 0). To understand the consequences of this displacement of the bifurcations, suppose that the coupling strength *σ* is positive and that the master subsystem is initially on its lower stable branch (the blue curve in figure 1*b*). Thus, *x*(0) = x* < 0 and σx* < 0, so the master subsystem suppresses a regime shift in the slave subsystem, meaning that the parameter *b* must increase further to pass *b*_break_(σx*) compared with the case of no coupling (*σ* = 0). However, if the master subsystem passes its break point (i.e. if *a* increases past *a*_break_), then the master subsystem *x*(*t*) jumps to its upper stable branch (the red curve in figure 1*b*), where *x*(*t*) = x* > 0. That sudden change facilitates a regime shift in the slave subsystem, meaning that the parameter *b* does not need to increase as much (in order to pass *b*_break_(σx*) = *b*_break_(0) − σx*) as it would if there were no coupling.

This simple system illuminates how regime shifts might synchronize. When the slow parameter *a* of the master subsystem (equation (2.2*a*)) increases past its saddle-node bifurcation at *a*_break_, the master subsystem jumps to its upper stable branch of equilibria (recall figure 1*b*), so the relevant saddle-node bifurcation for the slave subsystem (equation (2.2*b*)) suddenly changes from the blue curve to the red curve in figure 2*b*. Thus, at the moment when *a* passes *a*_break_, if the value of *b* lies above the red curve in figure 2*b* (and below the blue curve, meaning that the slave subsystem has not already jumped to its upper branch of equilibria), then the regime shift in the slave subsystem occurs *simultaneously* with the regime shift in the master subsystem. The green line segment in figure 2 marks the ‘synchronizing window’ *S*, the interval of values of (*a,b*) leading to synchronized regime shifts.^1^

For an interpretation of the synchronizing window, consider two economies *X* and *Y* that are both stuck in recession in the Kaldor business cycle model [13,18]. If *X* undergoes a boom, does the rise in the demand of *X* for imports from *Y* push *Y* out of its recession? The synchronizing window *S* specifies how close to its tipping point *Y* must be for the economic booms to synchronize, which provides an answer to Krugman's conjecture in [13].

In summary, there are three ways in which the two subsystems in equation (2.2) could both pass their breaking points, *a*_break_ and *b*_break_(σx*). First, the slave subsystem could undergo a regime shift on its own, meaning that *b* increases past the blue curve in figure 2*b* while *a* remains below *a*_break_, and subsequently *a* passes *a*_break_. Second, the two subsystems could simultaneously pass their breaking points, meaning that (*a,b*) crosses the synchronizing window in figure 2*b*. Third, the master subsystem could pass its breaking point *a*_break_, but the slave subsystem remains too far from its tipping point (despite becoming abruptly closer), so there is a delay in time between the regime shifts.

As the subsystems become more strongly coupled (larger coupling strength *σ*), it becomes easier for the regime shifts to synchronize: the S-shaped curves in figure 2*b* stretch vertically (but the intersections of the dashed curves and the *a* = 0 axis remain fixed), so the synchronizing window *S* enlarges with *σ*. (For an illustration, see the electronic supplementary material, figure SM-1.) The results of other simple coupling functions, such as ±*σ*|*x*|, are simple transformations of figure 2*b* (see electronic supplementary material, figure SM-2); we chose the coupling *σx* for simplicity. The results of this subsection also apply to couplings that form a directed star graph.^2^ (A glossary of terminology for graphs and networks is provided in appendix A.)

If the coupling were bidirectional, then the equilibria (x*, y*) would no longer be solvable in closed form. Although synchronized regime shifts could still occur, characterizing the equilibria becomes more complicated, as illustrated by Abraham's numerical studies [45]. (For more details on the related mathematical literature, see appendix B.) Next, we generalize in a way such that the equilibria remain analytically solvable.

### Master–slave–slave system 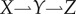


2.3.

Now we introduce a third subsystem *Z*, and we assume that *Y* drives *Z* in the same way in which the master subsystem *X* drives *Y* (and with the same coupling strength *σ*, for simplicity). Thus, we augment equations (2.2*a*) and (2.2*b*) with the equation
2.3
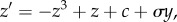
with a new slow parameter 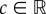 that, like *a* and *b*, changes much more slowly than *x*, *y* and *z* do.

Regime shifts can spread in two ways in this system 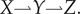 First, if all three systems are sufficiently close to their tipping points, then a cascade of regime shifts can occur, one causing the next. The second way is more novel: if the intermediate system *Y* is relatively far from its tipping point whereas *X* and *Z* are close to their tipping points, then the sequence of regime shifts can ‘hop’ over the intermediate system *Y*. That is, a regime shift in the master subsystem (equation (2.2*a*)) can nudge the intermediate system *Y* (equation (2.2b)) enough to trigger a regime shift in the third system *Z* (equation (2.3)) but not so much that *Y* undergoes a regime shift.

**Figure 3.**
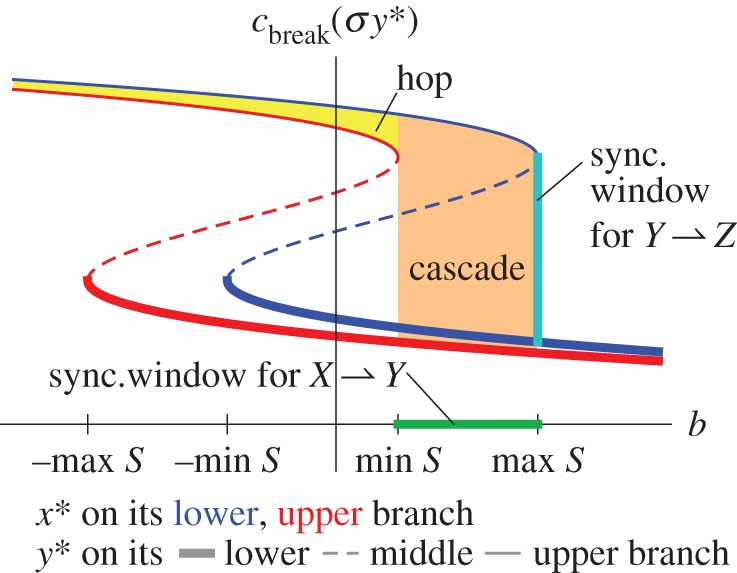
Catastrophes can cascade, or they can hop over intermediate systems. The two backward-S-shaped curves are plots of the break point *c*_break_(σ y*) of the downstream system *Z* (equation (2.3)) as a function of the slow parameter *b* of the intermediate system (2.2*b*) for coupling strength *σ* = 0.2. The two curves show the effect of the master parameter *a* increasing past its break point *a*_break_, at which time we change focus from the blue, right-hand curve to the red, left-hand curve. Thick curves (respectively, thin curves, dashed curves) correspond to the intermediate system y* on its upper stable branch (respectively, lower stable branch, middle unstable branch). As in figure 2*b*, the green line segment marks the synchronizing window *S* for *X* and *Y* (the values of *b* such that, when *a* crosses *a*_break_, the intermediate system *Y* passes its break point *b*_break_(*σ x* *)). The cyan line segment marks the analogous interval for *Y* and *Z* (for *a* = *a*_break_). The orange and yellow regions are values of (*b,c*) leading to sequences of regime shifts that cascade or that jump over *Y*, respectively.

We illustrate these two phenomena in figure 3, a plot of the ‘downstream subsystem’ *Z*'s break point *c*_break_(σy*) at the moment when the master subsystem's parameter *a* increases past its break point *a*_break_. At this moment, the master subsystem jumps from its lower branch of equilibria to its upper branch, so we change focus from the red curve to the blue curve in figure 3. If the slow parameters (*b,c*) lie in the orange region labelled ‘cascade’ in figure 3, then a cascade of regime shifts occurs, one regime shift causing the next. To see why, note that *b* lies in its synchronizing window *S* (the green line segment in figures 2*b* and 3), so *Y* passes its break point *b*_break_(σx*); and note that *c* lies above the thick, red line *c*_break(__*σ*y__*)_, so *Z* passes its break point *c*_break(__*σ*y__*)_. If, on the other hand, the parameters (*b,c*) lie in the yellow region labelled ‘hop’ in figure 3, then the sequence of regime shifts hops over the intermediate subsystem *Y*. To see why, note that *b* is below its synchronizing window *S*, so *b* does not pass its break point *b*_break(__*σ*x__*)_ when *a* crosses *a*_break_, but note that *c* lies above the red thin line, so *Z* passes its break point *c*_break(__*σ*y__*)_ despite receiving only a small nudge from *Y*.

Note that such ‘cascade hopping’ cannot occur in many classic models of cascades, including the Ising model [63], sandpile models [63] and threshold models [64,65]. For cascade hopping to occur, some vertices of the graph must be able to affect their neighbours in at least three ways (e.g. with a small, medium or large amount of force). A phenomenon that is qualitatively similar to cascade hopping occurs in epidemiology: some diseases are contagious yet asymptomatic, so the sequence of contractions of the disease can appear to hop over individuals. Different people remain in the asymptomatic state for different amounts of time, which resembles coupled subsystems with different proximities to tipping points.^3^ Next, we show circumstantial evidence that cascade hopping may occur in other kinds of contagion in human populations.

## 3. Communication-coupled outbreaks of protest

We have presented a ‘model of many models’ that captures a commonality among systems in table 1 but that ignores many domain-specific details. If the models in table 1 are one step removed from reality, then the stylized model in §2 is two steps removed from reality. The virtue of studying such a simple model is to elucidate what phenomena might happen in more realistic settings.

To give one example, in this section we consider protests and revolutions occurring in many countries. In §3.1, we summarize Kuran's model [30] of protests and revolutions based on preference falsification and Slee's model [31] of identity-driven cascades. Our model is a stylized generalization of these models to multiple countries, with finance and cross-border identity being two possible mechanisms for coupling protests across borders. Next, we study data on countries involved in the Arab Spring, the uprisings in Northern Africa and in the Middle East during 2010–2011. Using the theoretical model of §2 as a guide for asking questions, we explore the role of contagion and common cause in the Arab Spring (§3.2), whether protests seem to spread locally (§3.3) or in non-local jumps (§3.4).

### 3.1. Models of revolutions based on preference falsification and identity

To begin, we summarize two models of protests and revolutions that emerge suddenly via saddle-node bifurcations. Then we explain how the conceptual framework in §2 can capture a generalization of these models to multiple countries with couplings between them. (Not all models of protests have saddle-node bifurcations. For some recent examples, see [67–70].)

One way in which protests and revolutions can emerge suddenly is because people had been publicly declaring a preference different from their private preference [30]. This idea, called preference falsification, has been used in several applications [71]. In Kuran's model of revolutions [30], the unit interval [0, 1] denotes a political spectrum, with 0 representing the current government and 1 representing the opposition. He assumes that people derive ‘reputational utility’ from publicly declaring a certain preference in [0, 1], plus an ‘integrity utility’ from declaring a preference close to their private preference. Slow changes in these utility functions or in the distribution of preferences can cause a large, sudden change in collective sentiment (in a saddle-node bifurcation).

**Figure 4.**
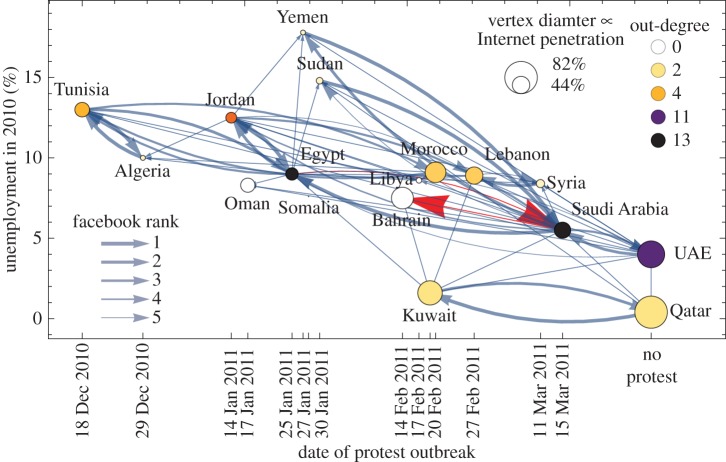
Exploration of the roles of proximities to tipping points and coupling (cross-border communication) for countries involved in the Arab Spring. Shown is the unemployment in 2010 [77] versus the date at which protests began [80] (table 3) for 16 countries that had protests, plus Qatar and the UAE. The best-fit line has slope −0.5% per week (*p*-value 0.06, *R*^2^ = 0.25; UAE and Qatar ignored). The direction of the Facebook edges [79] captures the spread of influence to protest: an edge from *i* to *j* means that *i* is among *j*'s five countries with which *j* has the most Facebook friends (in the year 2012); edge thickness decreases linearly with rank, so the thickest edges correspond to rank one (the strongest coupling). Vertex colour denotes out-degree; high out-degree nodes (e.g. Egypt and Saudi Arabia) may be particularly influential in spreading influence to protest via Facebook. Highlighted in red edges with large arrowheads is one cascade hop motif, Egypt → Saudi Arabia → Bahrain (see definition 3.1 for the definition and table 2 for the other nine hop motifs). A version of this plot with a few more countries that protested much later than the dates shown here is in the electronic supplementary material, figure SM-3.

Kuran's model is more rich than the model in §2, as it has utility functions, distributions of preferences and weights of different people, but the manifold of equilibria in Kuran's model is equivalent (in a catastrophe-theoretic sense [35]) to that of the isolated subsystem in equation (2.2*a*). The state variable in Kuran's model is the (weighted) share of people who publicly declare that they prefer the opposition. The equilibrium [30, eqn 8] has one or three equilibria; in the latter case, two equilibria are stable and the other unstable, as illustrated in [30, figs 3–7]. The difference between the thick and thin curves in figs 3–7 of [30] is the analogue of figure 1*a*.

Kuran explains two ways in which a saddle-node bifurcation can occur, leaving only one equilibrium corresponding to a large public support of the opposition [30, §4.1, pp. 51–53]:

(1) a shift in the distribution of private preferences toward the opposition [30, fig. 3] due to, for example, an economic downturn [72];
(2) a change in the reputational utility terms (for example, because the opposition becomes better able to give reputational utility), causing a shift in the threshold function that marks whether someone supports the opposition or the government.

These two shifts correspond to changes in a ‘slow variable’ (such as the variable *a* in equation (2.2*a*)).

To suggest that our model might capture a generalization of Kuran's model to multiple countries, we must motivate the assumption that the state variables in different countries are coupled somehow. Kuran mentions one possible mechanism: a shift in the reputational utility (item 2 in the list above) could be ‘made possible by funds provided by a foreign source’ [30, p. 53]. That is, a coupling to a foreign country (here, a financial type), could change the equations of motion such that two equilibria vanish, leaving only the equilibrium that corresponds to large support for the opposition. To continue Kuran's story, suppose that those foreign funds were sent from the opposition in a country that has just undergone massive protests, say, because that country passed a saddle-node bifurcation. This example corresponds to the master subsystem crossing its break point (*a* passes *a*_break_), and the coupling *σx* qualitatively captures the increase in the ability of the opposition in the second country to give reputational utility to supporters because of financial funds from abroad.

Identity provides another possible coupling across borders. Gause [32] argues that pan-Arab identity is an important reason why the Arab Spring protests emerged nearly simultaneously and why it took Middle East specialists by surprise. Identity that spans borders could couple decisions to protest. For example, in Slee's model of revolutions based on rational-choice theories of identity [31], people suffer disutility due to cognitive dissonance whenever their actions differ from the norms associated with their identity. Slee considers two identities associated with the government and with the opposition. Like in Kuran's model [30], small changes can eliminate two equilibria, causing large protests to erupt. To continue Slee's reasoning [31], if people protest in one country, then it becomes more important for others in a nearby country to act according to their anti-government identity. If *x*(*t*) measures the share of people in one country who are protesting, then the importance of identity in the utility functions of people in a different country could vary directly with *x*(*t*), such as the simple linear coupling 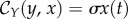 studied in §2.2 and §2.3.

These social-scientific models of revolutions based on preference falsification and identity illustrate how difficult it is to validate our coupled-threshold model with real data: these models are based on cognitive dissonance, preferences and identity. In principle, these cognitive phenomena could be studied with surveys, ethnographies and other labour-intensive methods. These theories [30,31], which are grounded in social scientific understanding of human behaviour, can be seen as the connection between our conceptual model and real systems. When we describe our model as a ‘model of models’ and hence two steps removed from reality, we have in mind models like [30,31] that have bistability.

Multiple equilibria can also arise if people have greater incentives to protest as more people decide to protest (i.e. strategic complementarities) [73], for example, because of safety in numbers [74, p. 18]. Multiple equilibria also occur in repeated coordination games in which people learn about the number of protestors needed to overthrow the regime (so-called dynamic global games) [75].

Now that we have connected the stylized model in §2 and the sociological literature such as [30,31], we next investigate data from the Arab Spring with questions generated from the conceptual framework of §2.

### 3.2. Contagion versus common cause in the Arab Spring

Why did many protests begin nearly simultaneously in the Arab Spring? One explanation is *common cause* (called the *monsoonal effect* in the context of contagious currency crises [20]): an external driver, such as rising global food prices, pushes all countries past their tipping points (as suggested in [76]). Another explanation is *contagion*: couplings among countries (such as communication) helped to synchronize their protests. The analogue of common cause in System (1) in §2 is that the slow parameters *a* and *b* both increase and pass their tipping points simultaneously (or at nearly the same time), with or even without coupling. The analogue of contagion is that the slow parameter *a* increases past its tipping point, which (via the coupling) pushes *b* past its tipping point.

To begin to explore the possible roles of common cause and contagion, we study data on attributes of countries [12,77,78] and data on communication between countries via Facebook and telephone. Communication across borders spread inspiration to protest, freedom memes and strategies for success [11,12,33]. Therefore, cross-border communication via Facebook and telephone may have spurred people to publicly declare their private preferences [30] or to act according to norms of their government-opposing identities [31]. The Facebook data available [79] are coarse-grained: for each country, we have the ranked list of the top five other countries to which members of the focal country have the most friends (in 2012, the only year available to us).

Figure 4 shows the subgraph of this Facebook graph induced by countries that protested in the Arab Spring, together with two countries that did not protest but that may have communicated influence to protest and that shared Arab identity, Qatar and the United Arab Emirates.^4^ Next, to explore the possible roles of common cause and contagion, we study what attributes of countries correlate with when their protests began. We found that unemployment most significantly correlates with protest start date (see the downward trend in figure 4). That suggests that high-unemployment countries were closer to their tipping points.^5^

Internet penetration, the fraction of the population that uses the Internet [77], which is plotted as vertex diameter in figure 4, may indicate the strength of coupling to other countries via social media such as Facebook, which was thought to be an important channel for inspiring protests [11,34]. However, Internet penetration is a weak and statistically insignificant predictor of when protests started in various countries.^6^

Spikes in commodity food prices have been proposed as a significant cause of the Arab Spring [76]. Here we consider consumer food prices [78], which did not notably spike in 2010 [81]; we found that these indices in 2010 were not predictive of when protests began in different countries.^7^

Many other covariates, from economic indicators to political freedoms, were similarly weak and statistically insignificant predictors of when protests began (for the list of covariates, see the first column of electronic supplementary material, figures SM-4 and SM-5). Furthermore, using the criterion for forward selection, we could not reject the null hypothesis that any of these covariates could be considered together with unemployment.

Because these network data are longitudinal, hazards models [82] or generalized estimating equations [83] could be useful. A challenge, however, is the small sample size (about a dozen countries).

### 3.3. Did Arab Spring protests spread locally?

The ‘domino hypothesis’—that the Arab Spring protests spread locally like falling dominoes—has been the subject of speculation [12,84,85] but, to our knowledge, little analysis. An alternative hypothesis, motivated by the ‘hopping’ phenomenon in §2.3, is that Arab Spring protests spread non-locally in some way.

We find circumstantial evidence in support of both hypotheses. In support of the domino hypothesis, we found that, among countries that had protests, a majority of those countries share a border^8^ with at least one country whose protests began earlier, and a majority has a Facebook link from at least one country whose protest began earlier. These results are statistically significant compared with a null model of randomized protest dates.^9^ In addition to this evidence of local spread, we also find evidence of protests spreading non-locally, discussed next.

### 3.4. Cascade hopping in the Arab Spring

Here we show circumstantial evidence that protests may have spread in a non-local way consistent with the ‘cascade hopping’ phenomenon in §2.3. An empirical signature of the ‘hopping’ phenomenon—though not conclusive evidence of it—is a small subgraph in which protests appear to hop over a country. If this small subgraph appears more often compared with a reasonable null model, then this subgraph is called a ‘motif’. We call this particular motif a ‘hop motif’ and define it as follows. (For definitions of network terminology, see the Glossary in appendix A.)

**Definition 3.1.**

A hop motif in a directed coupling graph is a triple of countries (*X*, *Y*, *Z*) such that

(1) the subgraph induced by {*X*, *Y*, *Z*} is the directed path 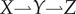;^10^
(2) there is no coupling edge pointing to *Z* from any country that began to protest before *Z* did; and
(3) protests began first in *X*, then in *Z*, and then in *Y* (or *Y* did not have any protests).

For the Facebook network shown in figure 4, 10 triples of countries, listed in table 2, satisfy these criteria in definition 3.1. One of them, Egypt → Saudi Arabia → Bahrain, is highlighted with red edges and large arrowheads in figure 4. Compared with a null model with random protest start times, the network in figure 4 has more hop motifs than 93.3% of randomized versions.

**Table 2.** Ten ‘hop motifs’ in the Facebook data (see definition 3.1). The notation ‘
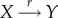
’ means that country X is located at position *r* on the list of countries ranked in descending order by the number of Facebook friends with people in country Y. For example, ‘Egypt 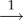 Saudi Arabia’ means that Saudis have more Facebook friends in Egypt than in any other country.

upstream *X* 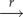 intermed. *Y* 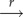 downstream *Z*
Egypt 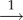 Saudi Arabia 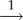 Bahrain
Yemen 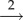 Saudi Arabia 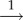 Bahrain
Tunisia 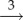 Egypt 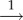 Jordan
Jordan 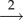 Egypt 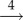 Oman
Tunisia 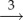 Egypt 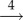 Oman
Egypt 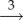 Kuwait 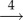 Bahrain
Jordan 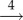 Saudi Arabia 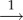 Bahrain
Jordan 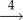 Saudi Arabia 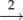 Oman
Sudan 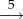 Saudi Arabia 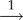 Bahrain
Egypt 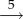 UAE 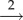 Bahrain

These hop motifs suggest (but do not conclusively show) that Saudi Arabia and Egypt played the role of an intermediate subsystem *Y* in §2.3. Specifically, the motifs suggest that Saudi Arabia and Egypt may have received influence from protesting countries that played the role of the upstream subsystem *X* (e.g. Tunisia, Jordan) and propagated influence to other countries that played the role of the downstream subsystem *Z* (e.g. Bahrain, Oman), which may have helped to trigger protests in *Z* before protests began in *Y*. Consistent with relative deprivation theory (which argues that economic stress puts countries close to a tipping point) [72], we find that the upstream and downstream countries in table 2 were relatively closer to tipping points than intermediate countries (see the electronic supplementary material, §SM-4).

Unlike work on temporal motifs in telephone call data [87,88], here events occur on the nodes rather than on the edges (i.e. protests occur in countries, whereas phone calls occur between individuals). Thus, hop motifs were not studied in work on temporal motifs [87,88].

Note that a hop motif (*X*, *Y*, *Z*) is delicate: a communication link from *X* to *Z* could explain why protests began in *Z* before they began in *Y*. None of the upstream and downstream countries *X* and *Z* share a border, and only Jordan and Oman had a significant amount of cross-border telephone calls in 2010 (8.3 × 10^6^ min), which eliminates two of the 10 hop motifs in the Facebook network (table 2). Data on other communication between countries, such as cross-border mentions of hashtags on Twitter [11] and consumption of news media, could reveal communication from *X* to *Z*, but obtaining such data are difficult and beyond the scope of this paper. A limitation of the Facebook dataset is that we only know the top-5 countries to which each country has the most Facebook friends; considering only the top-*R* lists with 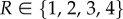 did not result in any new hop motifs.

## 4. Discussion

Some of the most pressing global challenges involve the prediction and control of sudden changes propagating among coupled subsystems, such as avoiding disastrous shifts in the biosphere [1] and preventing crises in the financial system [10]. Livelihoods could also improve if sudden adoption of technologies in coupled markets were facilitated [13,14], or if coupled recessions and booms in economies were better managed [5–8,13], or if social uprisings spreading among countries were better forecast [11,12,32]. Mathematically understanding tipping points in coupled subsystems is a step toward meeting these challenges.

In this paper, we have shown in a conceptual model how regime shifts can propagate among coupled subsystems by cascading or even by jumping over subsystems. Here, we model a regime shift as a parameter passing a saddle-node bifurcation, which causes a sudden change to a different equilibrium. Such behaviour appears in many systems [89,90] but is not the only kind of regime shift [36,91]. This model combines continuous and discrete, threshold-like changes. The study of models with these features is a challenge in several disciplines, such as in failures spreading in economic input–output models [92] or in electric power grids (though in a more non-local way) [93,94]. We also find non-local spread: the next subsystem to pass a tipping point may lie two or more ‘hops’ away from those that have passed their tipping points.

This model captures just one aspect of many models (couplings and saddle-node bifurcations), but it ignores many domain-specific details that could also be quite important. At best, this ‘model of many models’ can suggest what phenomena might occur in more complicated, domain-specific models or in real data. As an example, we find 10 ‘hop motifs’ (i.e. sequences of sudden changes that appear to hop over intermediate subsystems) in data on communication among countries involved in the Arab Spring protests.

Much attention is devoted to regime shifts in large, central nodes, such as the effect of recessions in large economies or the question of whether to bail out large banks. Our findings suggest that small, seemingly innocuous changes in these central nodes (perhaps triggered by a large change in a small node adjacent to it) can suffice to trigger a regime shift in a peripheral node close to its tipping point. Such dynamics may have occurred in the aftermath of the 2008 financial crisis given that in the United States hundreds of small banks failed but few large banks failed [95]. Peripheral players in networks may be vulnerable to sequences of regime shifts that hop over the core, an issue that seems to merit further attention.

An open challenge is to estimate tipping points (if they exist at all) in various complex systems, using data from historical examples. Considering data not only from the Arab Spring but also from other episodes of nearly synchronous uprisings (e.g. in Soviet countries in 1989 [74,96,97] and others [98]) could elucidate how couplings among countries affect their proximities to tipping points. This understanding could enable better prediction of the next protest or revolt, complementing new techniques for mining news for sentiment and tone [99,100] and early warning signals applied to social network activity [101]. Similar advances have been made in understanding contagion of currency and debt crises among countries in the 1990s [20].

Another challenge is to extend work on temporal motifs in telephone call data [87,88] to settings like the one considered here. In the systems summarized in table 1 and in the model in §2, events occur on the nodes (rather than on the edges [87,88]), and nodes can be influenced by multiple ongoing events (rather than participating in just one event at a time [87,88]). Hop motifs are just one example in this new class of temporal motifs.

## Supplementary Material

CoupledCatastrophes_final_version_Supplement.pdf

## Figures and Tables

**Table 3.** Dates when protests, demonstrations or conflicts began in the Arab Spring.

country	date protests began
Tunisia	18 December 2010
Algeria	29 December 2010
Jordan	14 January 2011
Oman	17 January 2011
Egypt	25 January 2011
Yemen	27 January 2011
Djibouti	28 January 2011
Somalia	28 January 2011
Sudan	30 January 2011
Bahrain	14 February 2011
Libya	17 February 2011
Kuwait	19 February 2011
Morocco	20 February 2011
Mauritania	25 February 2011
Lebanon	27 February 2011
Syria	11 March 2011
Saudi Arabia	5 March 2011
Israel	15 May 2011
Palestinian Territory	4 September 2012
Iraq	23 December 2012

## Endnotes

1 Specifically, *S* is the Cartesian product {*a*_break_} × *I*, where  is the closed interval with the minimum (respectively, maximum) of *I* equal to the value of *b*_break_(*σx**) for *x** on its upper (respectively, lower) branch of equilibria at *a* = *a*_break_. The ‘break-type’ regime shifts synchronize if and only if the slow variables (*a, b*) pass through *S*.

2 A directed star coupling graph,  is a system with one master subsystem that evolves according to equation (2.2*a*) and that drives *n* − 1 slave subsystems according to equation (2.2*b*), with potentially different parameters for the various slave subsystems *Y_i_*.

3 HIV is an example of an infection with large variability in asymptomatic periods, resulting mostly from variability among patients [66]

4 Expatriates in other countries may be important, too, but we add only Qatar and the UAE to our analysis given their geographic and cultural proximity to the countries with protests.

5 Linear regression indicates that each additional 1% in unemployment in 2010 is associated with protests starting 3.2 ± 1.6 days earlier (mean ± 1 s.e.); the *p*-value is 0.06 and *R*^2^ is 0.25, suggesting statistical significance.

6 Each percentage of Internet users delays the protest start date by 0.44 ± 0.37 days; *p*-value 0.25, *R*^2^=0.099.

7 The index [78] is expressed on a scale such that it equals 100 in the year 2000. Each unit increase in the consumer food price index is associated with protests occurring 0.09 ± 0.26 days earlier, but with *p*-value 0.74 and *R*^2^=0.011.

8 We assume that Bahrain borders Saudi Arabia, Qatar and the UAE given the proximity and the fact that Saudi Arabia and the UAE sent troops to Bahrain to quell protests there [86], suggesting that protests Bahrain would likely spread to Saudi Arabia and the UAE.

9 Specifically, seven of the 16 countries with protests in figure 4 and 11 of all 20 countries with protests (listed in table 3) shared a border with at least one country with protests; comparing this outcome to randomized protest start times gives a *p*-value of 0.02 in both cases. For the Facebook graph, 10 of the 16 countries with protests in figure 4 and 14 of all 20 countries with protests (listed in table 3) had at least one incoming Facebook link to a country with protests that started earlier (*p*-values 0.06, 0.16, respectively).

10 For the Facebook graph [79], this path subgraph means that *X* is on *Y*'s top-5 list (of countries to which people in *Y* have the most friends) and that *Y* is on *Z*'s top-5 list.

## Data accessibility

The following data used in this article are freely available online:

— Unemployment in 2010, unemployment of young men (age 15–24) in 2010, GDP *per capita* in 2010 (based on purchasing power parity in constant 2011 US dollars) and Internet penetration in 2010 are from The World Bank [77].
— GDP *per capita* for Djbouti, Libya, Syria and Somalia were missing in the World Bank data [77]. To fill these gaps, we used the GDP *per capita* (for the year 2011) in the Wolfram|Alpha knowledgebase (http://www.wolframalpha.com/).
— Missing data on Internet penetration in 2010 for Somalia in the World Bank data [77] were filled using data from [102] by linearly interpolating the Internet use between 2009 (1.16%) and 2011 (1.25%), arriving at the estimate of 1.2%.
— The Gini coefficient of the wealth distribution, level of oil production (electronic supplementary material, figure SM-4), fraction of the population living in urban areas (electronic supplementary material, figure SM-5), fraction of the population under age 25 (electronic supplementary material, figure SM-5) and success of the protest in achieving its goals with minimal violence (electronic supplementary material, figure SM-5), are from ref. [12, table 1].
— Political freedom data (the fourth and fifth rows of electronic supplementary material, figure SM-5) are from Freedom House [103].
— Freedom of the press data (the last row of electronic supplementary material, figure SM-5) are from [104].
— The Facebook data [79] were scraped from a Facebook blog post.
— The cross-border telephone data [105] are from 2010. For each country, the data have the top 10 to 20 countries with the most outgoing telephone calls from the focal country, measured in millions of minutes. We only have data for pairs with at least a million minutes. These data were purchased from TeleGeography, and are available at http://spins.ucdavis.edu/.
— Table 3 gives the dates at which protests, demonstrations or conflicts began in the 20 countries that had some form of demonstration or conflict during the Arab Spring (from [80]). These dates are the horizontal positions of countries in figure 4 in the main text and in electronic supplementary material, figure SM-3.
— Shared borders were computed using the function *CountryData* in the Wolfram Language [106].

## Appendix A. Glossary of network terms

— A *network* (or *graph*) is a collection of *nodes* (or *vertices*) and a list of connections (or *edges*) among them. For example, in a social network, the nodes are people and the connections could be friendships. If those connections have a direction, then the graph is called *directed*; otherwise the graph is called *undirected*. Graphs are typically visualized by drawing the nodes as circles and the edges among them as lines; if the edges are directed, then the lines have arrowheads to indicate their direction.
— A node's *degree* is the number of connections it has. A node in a directed graph has an *in-degree* and an *out-degree*, which are the numbers of incoming and outgoing connections, respectively.
— A *subgraph* of a graph is a graph that is entirely contained in the original graph. A *subgraph induced by a certain subset of nodes* is the subgraph consisting of all the edges among those nodes.
— A *motif* of a graph is a small subgraph that appears rather frequently compared with some randomized version of the graph.

## Appendix B. Literature related to System (1)

The case of System (1) with bidirectional, symmetric coupling  is a special case of the double cusp catastrophe, which is given by the potential

This singularity has a very rich structure [41–43].

Abraham *et al*. [45] numerically studied a system similar to the double catastrophe, namely   which is System (1) but with no constant terms (i.e. *a* = *b* = 0) and with coupling functions  providing the only terms independent of *x* and independent of *y*, respectively. They numerically study the bifurcation sets by plotting the number of equilibria as a function of the four parameters . Abraham [44] also outlined how one might study this system with *n* equations coupled via some graph; our paper can be seen as an implementation of this idea.

The widely studied van der Pol oscillator   upon a Liénard transformation   becomes

B 1*a*

and

B 1*b*

Note that equation (B 1) has the same form as equation (2.1*a*) in the uncoupled case []. Equation (B 1*b*) is a differential equation for the parameter that plays the role of *a* in equation (2.1*a*). The van der Pol oscillator has a unique, stable limit cycle around the origin [46]. Many papers have studied coupled van der Pol oscillators, with a focus on stability of oscillations [46–49] and on chaos [50,51], many inspired by biological applications [46,48,52,53]. A related limit-cycle oscillator is the Fitzhugh–Nagumo model, a two-dimensional ODE that, when coupled to another such system, can produce chaos [107, §6.3.3]. Here, we focus on the contagion of regime shifts in the singular limit, which corresponds to the limit  in equation (B 1*b*).
