# Supplementary material for: Coupled catastrophes: sudden shifts cascade and hop among interdependent systems
Source: J R Soc Interface. 2015 Nov 6;12(112):20150712. doi: 10.1098/rsif.2015.0712 (PMC4685844; doi:10.1098/rsif.2015.0712)
Supplement: CoupledCatastrophes_final_version_Supplement.pdf [file rsif20150712supp1.pdf]

Coupled catastrophes: sudden shifts cascade and hop among interdependent systems
Supplementary Material

Charles D. Brummitt*[1,2,6], George Barnett[3], and Raissa M. D'Souza[2,4,5,7]

[1]Department of Mathematics, [2]Complexity Sciences Center, [3]Department of Communication, [4]Department of Computer Science, [5]Department of Mechanical Engineering, University of California, Davis, CA 95616 USA, [6]Center for the Management of Systemic Risk, Columbia University, New York, NY 10027 USA, [7]Santa Fe Institute, Santa Fe, NM 87501 USA

October 16, 2015

SM-1 Stronger coupling makes it easier to see synchronized regime shifts

If the coupling strength σ is increased, then, as Fig. SM-1 illustrates, the S-shaped curves in Fig. 2(b) are stretched vertically, which increases the length of the synchronizing window. In fact, one can calculate the synchronizing window S for System (2); the result is

$$S = \left[\frac{2\,(1-3\sigma)}{3\sqrt{3}}, \frac{2\,(1+3\sigma)}{3\sqrt{3}}\right],$$

which is illustrated in Fig. SM-1(c). The implication of this expression is that strengthening the coupling makes synchronized regime shifts more likely, in the sense that more paths in parameter space lead to synchronized regime shifts.

SM-2 Other simple couplings

The results for coupling functions $\mathcal{C}_Y(x,y) = \sigma x$ and $\mathcal{C}_X(y,x) = 0$ with $\sigma < 0$ can be obtained by reflecting the S-shaped curves $b_{\text{break}}(\sigma x^*)$ and $b_{\text{sustain}}(\sigma x^*)$ about the lines $b = b_{\text{break}}(0)$ and $b = b_{\text{sustain}}(0)$, respectively. In this case, the master subsystem facilitates (respectively, impedes) the slave subsystem's sudden shift to its upper branch of equilibria when x^* is on its lower (respectively, upper) branch of equilibria. The effects of coupling functions $\mathcal{C}_Y(x,y) = \sigma|x|$ can be obtained similarly. They are illustrated in Figure SM-2.

SM-3 Facebook subgraph induced by all countries with protests

In Fig. 4, we excluded Djibouti because it does not have unemployment data, and we excluded Israel, Palestinian Territory, and Iraq because their protests began much later (May 2011, September 2012, and

*Corresponding author: c.brummitt@columbia.edu

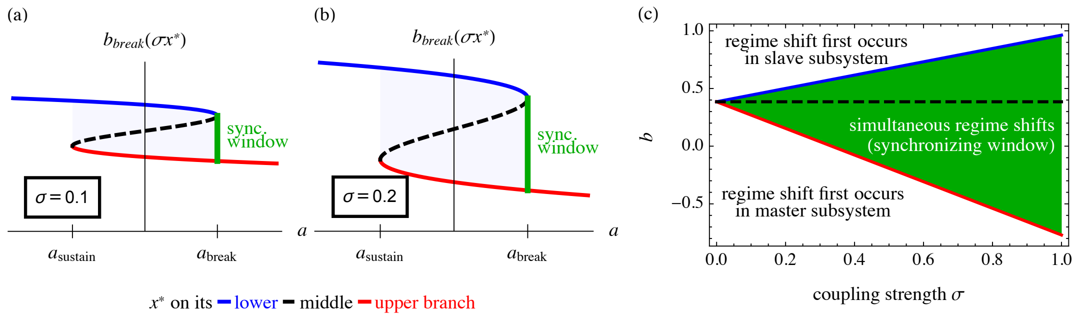

Figure SM-1: **Stronger coupling facilitates synchronization of regime shifts.** When a increases past a_{break}, a regime shift occurs simultaneously in the master–slave system (2) if and only if b [which is the slow parameter of the slave subsystem, Eq. (2b)] lies in the synchronizing window $S = \left[\frac{2}{3\sqrt{3}}(1-3\sigma), \frac{2}{3\sqrt{3}}(1+3\sigma)\right]$, depicted as a green line in panels (a) and (b) and as a green region in panel (c). Panels (a) and (b) show how the S-shaped curves $b_{\text{break}}(\sigma x^*)$ [i.e., the "break point" saddle-node bifurcation of the slave subsystem, Eq. (2b)] stretch vertically as the coupling strength σ is increased [$\sigma = 0.1$ in panel (a) and $\sigma = 0.2$ in panel (b)], thereby enlarging the synchronizing window. Panel (c) shows the synchronizing window S as a function of the coupling strength σ. If b is below (respectively, above) S when $a = a_{\text{break}}$, then the regime shift first occurs in the master (respectively, slave) subsystem, and there is a delay between regime shifts. The dashed line marks the value of $b_{\text{break}}(\sigma x^*)$ for the case in which the subsystems are uncoupled ($\sigma = 0$), namely, $2/(3\sqrt{3})$.

December 2012, respectively). We also excluded Mauritania because its unemployment was so large (31.1%) that including it in Fig. 4 would obscure the rest of the data.

Figure SM-3 replicates Fig. 4 with these countries excluded in Fig. 4. Note that Djbouti and Mauritania have very small Internet penetration (6.5% and 4%, respectively), and they participate little in the Facebook subgraph shown in Fig. SM-3 (Djibouti has in-degree 2 and out-degree 1, while Mauritania has in-degree 3 and out-degree 0). Thus, we do not expect that excluding them from Fig. 4 has significant effects on the results in Sec. 3.2.

SM-4 Properties of countries in the hop motifs

Figures SM-4 and SM-5 show properties of the countries in these different roles X, Y, Z in the hop motifs. The "upstream" countries X appear to be relatively close to their tipping points because of their relatively high unemployment, high economic inequality, low GDP per capita, and large youth bulges. Recall that intermediate countries Y may have spread influence to protest from these upstream countries X to "downstream" countries Z. Country Z began to protest before Y did, perhaps because Z was closer to its tipping point [e.g., downstream countries Z had significantly higher unemployment and greater economic inequality (Gini coefficient)] and because intermediate countries tend to have relatively strong economies and significant revenue from oil, indicating a large distance from a tipping point. Intermediate and downstream countries had significantly higher Internet and mobile phone penetration, indicating their greater susceptibility to influence from ongoing protests.

Political rights and civil liberties [1] are too coarse-grained to distinguish among these countries. However, some more specific measures, such as personal autonomy and individual rights, show greater variance among countries involved in the Arab Spring; the fifth row of Fig. SM-5 shows that intermediate countries Y (Kuwait, Saudi Arabia, Egypt; data for U.A.E. is missing) tend to have greater personal autonomy and individual rights, which may play a role in delaying their protests. However, even when these populations were not yet protesting, these citizens may nevertheless have been communicating inspiration to protest to

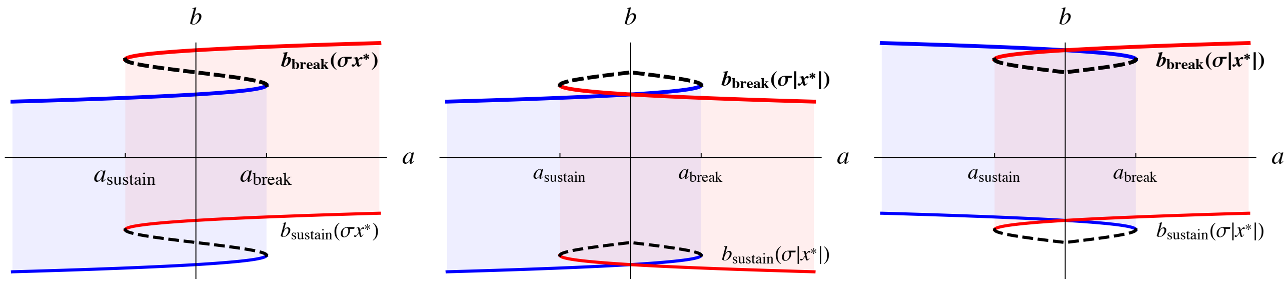

Figure SM-2: **Effect of other couplings on the slave subsystem's bifurcation diagram.** As in Fig. 2(b), if the master subsystem's equilibrium x^* lies on its lower (respectively, upper) stable branch depicted in Fig. 1(b), then the saddle-node bifurcations of the slave subsystem, $b_{\text{break}}(\sigma x^*)$ and $b_{\text{sustain}}(\sigma x^*)$, are the blue (respectively, red) curves; the dashed curves correspond to the master subsystem being on its middle, unstable branch of equilibria. If there were no coupling, i.e., if σ were equal to 0, then the saddle-node bifurcations $b_{\text{break}}(\sigma x^*)$ and $b_{\text{sustain}}(\sigma x^*)$ would be given by the intersections of the black-dashed curves and the $a = 0$ axis; comparing the blue and red curves with these intersections determines whether the master subsystem facilitates or inhibits a regime shift in the slave subsystem. Panel (a): The coupling $\mathcal{C}_Y(x, y) = \sigma x$ with $\sigma = -0.1$ makes it more difficult for the slave subsystem to cross its break point $b_{\text{break}}(\sigma x^*)$ when the master subsystem has crossed its break point ($x^* > 0$), and vice versa. Panel (b): The coupling $\mathcal{C}_Y(x, y) = \sigma|x|$ with $\sigma = 0.1$ makes it easier for the slave subsystem to cross its break point $b_{\text{break}}(|\sigma x^*|)$ no matter the sign of x. However, once the slave subsystem has crossed its break point, the value of b must be reduced considerably more in order to cross the "sustain" saddle-node bifurcation, $b_{\text{sustain}}(|\sigma x^*|)$, because of the coupling $\sigma|x|$ with $\sigma > 0$. Flipping the sign of σ, as shown in panel (c), reverses these effects.

other countries. Consistent with this interpretation, these intermediate countries also had greater freedom of the press (bottom row of Fig. SM-5).

References

[1] Freedom House. Freedom in the World: Aggregate and Subcategory Scores, 2010. Subcategory scores retrieved on September 12, 2014 from `http://www.freedomhouse.org/report/freedom-world-aggregate-and-subcategory-scores#.VBMdiC5dUuG`.

[2] M. M. Hussain and P. N. Howard. What Best Explains Successful Protest Cascades? ICTs and the Fuzzy Causes of the Arab Spring. *International Studies Review*, 15(1):48–66, Apr. 2013. ISSN 1468-2486. doi:10.1111/misr.12020.

[3] The World Bank, 2010. URL `http://data.worldbank.org`. Accessed May 14, 2014.

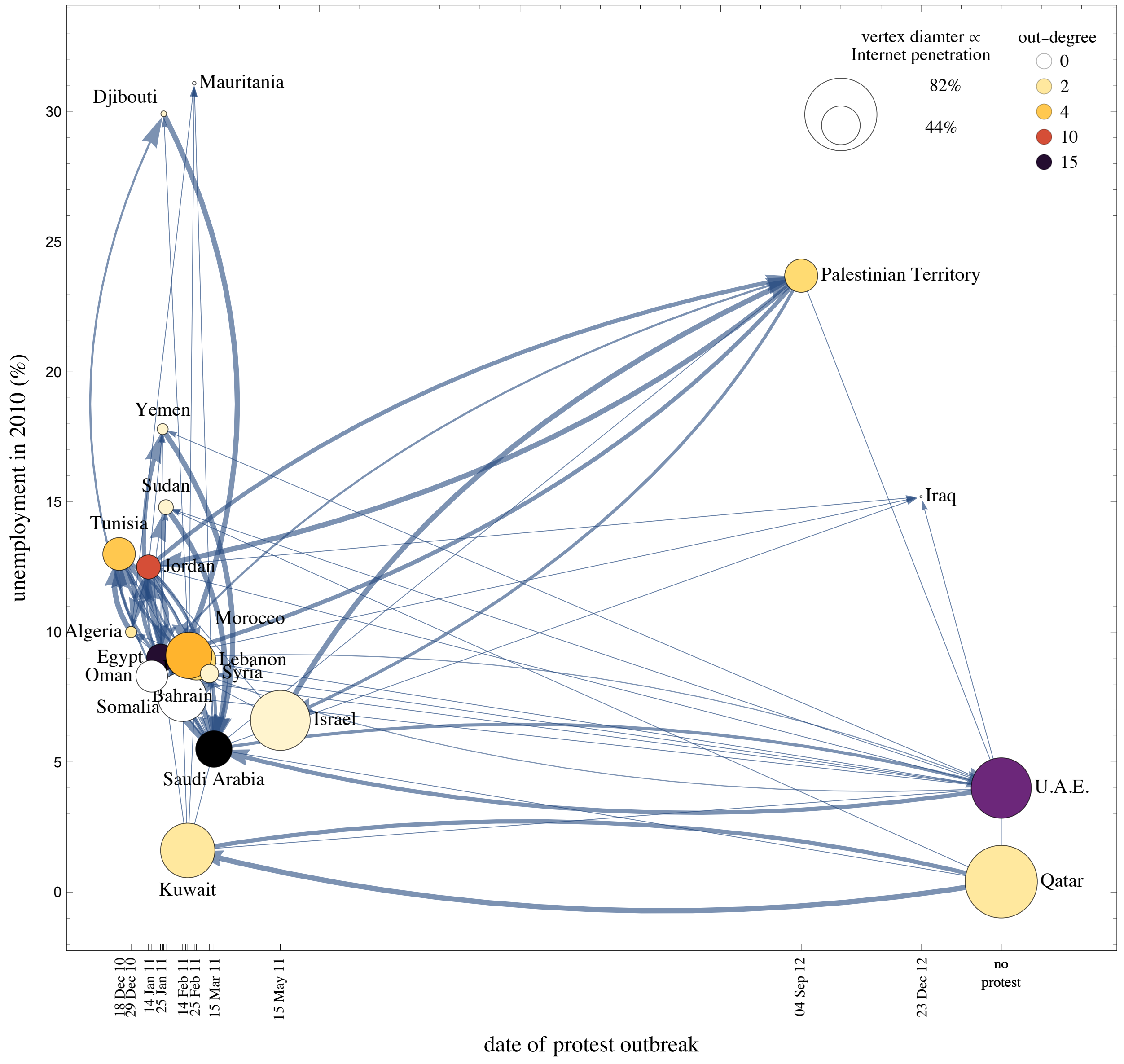

Figure SM-3: Facebook graph with countries excluded from Fig. 4 (namely, Djibouti, Israel, Palestinian Territory, Iraq, and Mauritania). The 2010 unemployment rate for Djibouti (30%) was estimated from a linear regression between the available World Bank data [3] and the "fuzzy" data in Table 1 of [2] (in which they estimate missing data by comparison with similar countries).

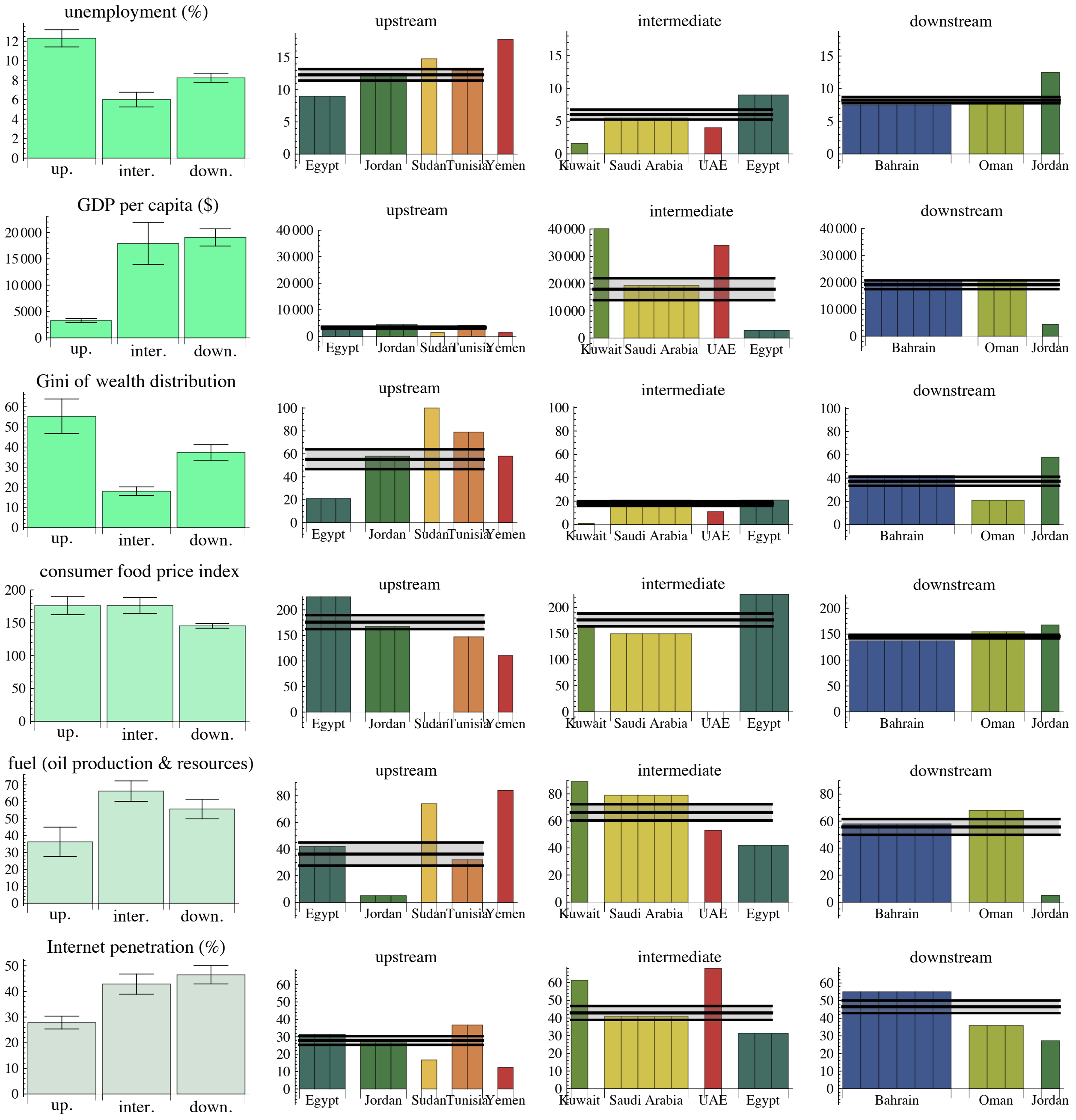

Figure SM-4: **Economic properties of countries in the ten hop motifs** (Table 2). Each row is one attribute of a country (unemployment, etc.) The left-hand column are weighted averages of the countries in the different roles X, Y, Z (labeled upstream, intermediate, downstream) in the hop motif (see Definition 1). The right-hand three columns show the countries in each of those roles; in each of these plots, the thick, horizontal line shows the mean, while the thin lines show the mean ± 1 standard error. All data are from 2010.

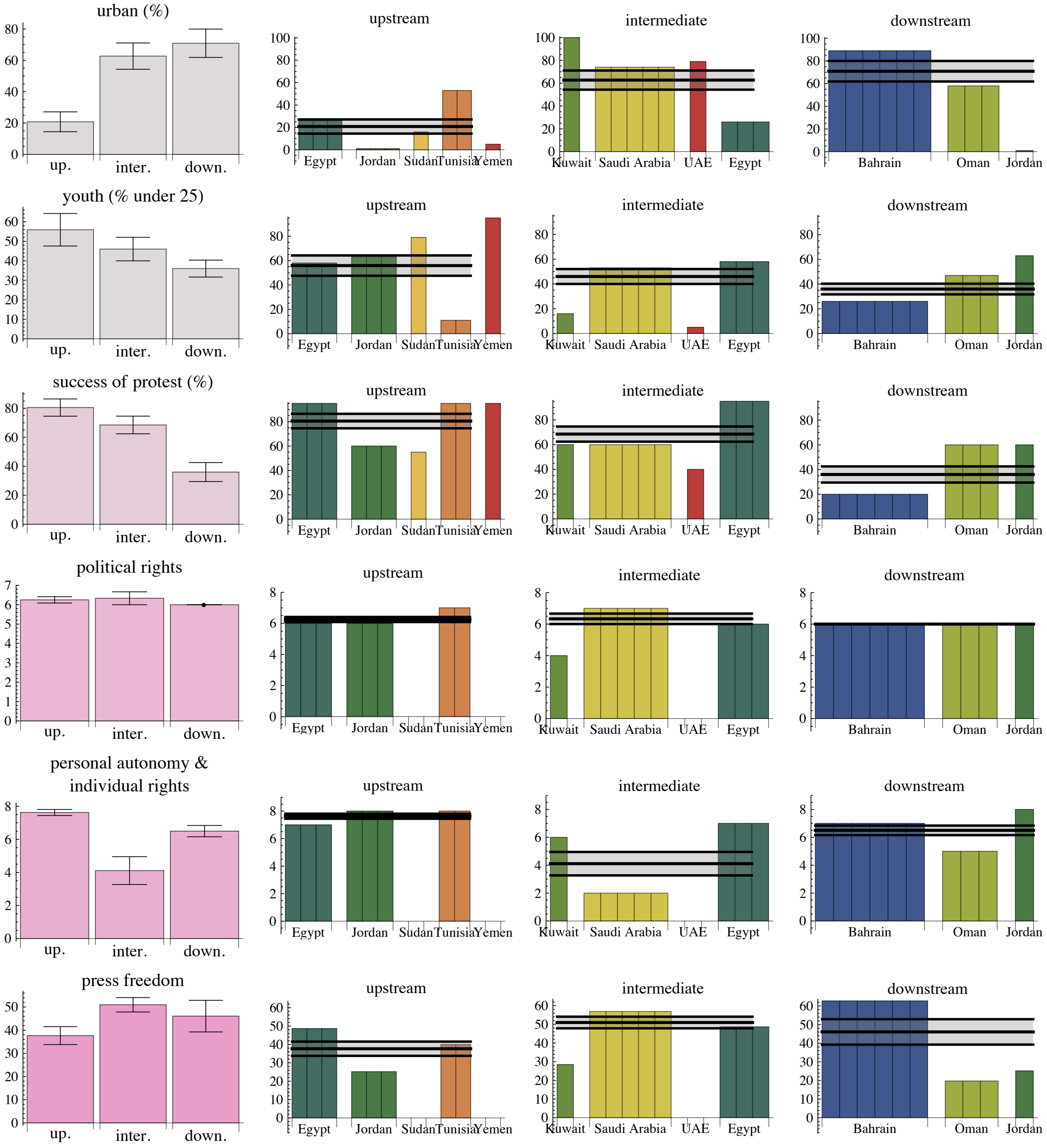

Figure SM-5: **Social and political properties of countries in the ten hop motifs** (Table 2). As in Fig. SM-4, the left-hand column shows weighted averages for the countries in the three roles X, Y, Z in the hop motifs (as defined in Definition 1), while the other columns show the countries (and their properties) in those weighted averages. All the data are from 2010 except for press freedom (2013).
